# Supplementary material for: Reconciling Mining with the Conservation of Cave Biodiversity: A Quantitative Baseline to Help Establish Conservation Priorities
Source: PLoS One. 2016 Dec 20;11(12):e0168348. doi: 10.1371/journal.pone.0168348 (PMC5173368; doi:10.1371/journal.pone.0168348)
Supplement: S1 Dataset — (ZIP) [file pone.0168348.s002.zip › Taxa/Serra Norte/SN_2013/N5S/Lista N5S-41.pdf]

CAVIDADE N5S-0041

Classe	Ordem	Fam/Outros	Gên/Outros	Espécie	S-U	U-S	Zon.
Arachnida	Araneae	Ctenidae		jovem		1	E
Arachnida	Araneae	Drymusidae		jovem	X		E
Arachnida	Araneae	Mysmenidae		jovem		X	E
Arachnida	Araneae	Pholcidae		jovem	X	X	E
Arachnida	Araneae	Salticidae		sp.11	X	X	E
Arachnida	Araneae	Segestriidae		jovem	X		E
Arachnida	Araneae	Theridiidae		jovem	X		E
Arachnida	Araneae	Theridiosomatidae	*Plato*	sp.1		X	E/P
Arachnida	Palpigradi	Eukoeneniidae	*Eukoenenia*	sp.1	X		E
Arachnida	Pseudoscorpiones	Chernetidae	*Spelaeochernes*	sp.1	X		E
Arachnida	Pseudoscorpiones	Chthoniidae		jovem		X	E
Chilopoda	Scutigeromorpha	Pselliodidae	*Sphendononema*	jovem	1		E
Entognatha	Collembola	Entomobryidae		sp.1	X		E
Entognatha	Collembola	Paronellidae		sp.1		X	E
Entognatha	Collembola	Paronellidae		sp.2		X	E
Entognatha	Diplura	Campodeidae		sp.1		X	E/P
Insecta	Blattodea	Polyphagidae		sp.1	2	3	E/P
Insecta	Coleoptera			jovem	X	X	E/P
Insecta	Diptera	Cecidomyiidae	Cecidomyiinae	sp.		X	E/P
Insecta	Diptera	Chloropidae		sp.	X		E
Insecta	Diptera	Ephydridae		sp.		X	E
Insecta	Diptera	Psychodidae	Phlebotominae	sp.		X	E/P
Insecta	Heteroptera	Dipsocoroidea		sp.	X		E
Insecta	Heteroptera	Nabidae		sp.		X	P
Insecta	Heteroptera	Reduviidae	Reduviinae	sp.	3		E
Insecta	Homoptera	Cixiidae		sp.5	X	X	E
Insecta	Hymenoptera	Chalcidoidea		sp.1		X	E
Insecta	Hymenoptera	Formicidae	*Camponotus*	sp.2		X	E/P
Insecta	Hymenoptera	Formicidae	*Dolichoderus*	aff. *bispinosus*	X	X	E/P
Insecta	Hymenoptera	Formicidae	*Pheidole*	sp.4	X		E
Insecta	Isoptera	Termitidae	*Nasutitermes*	sp.	X	X	E/P
Insecta	Lepidoptera	Gelechioidea		sp.		X	E/P
Insecta	Lepidoptera	Noctuoidea		sp.		2	E
Insecta	Neuroptera	Myrmeleontidae		jovem		X	P
Insecta	Orthoptera	Phalangopsidae	*Paraclodes*	jovem	3		E
Insecta	Orthoptera	Phalangopsidae	*Phalangopsis*	sp.		21	E
Insecta	Psocoptera	Psyllipsocidae	*Psyllipsocus*	sp.3	X	X	P
Insecta	Thysanura	Nicoletiidae		sp.1	X		E
Onychophora	Onychophora	Peripatidae	*Peripatus*	sp.1	1		E
Amphibia	Anura	Strabomantidae	*Pristimantis*	cf. *fenestratus*	2	2	E/P
